# Human Placental Mesenchymal Stem Cells for the Treatment of ARDS in Rat

**DOI:** 10.1155/2022/8418509

**Published:** 2022-06-22

**Authors:** Zurab Kakabadze, Nicholas Kipshidze, Teona Paresishvili, Nodar Kipshidze, Zurab Vadachkoria, David Chakhunashvili

**Affiliations:** ^1^Department of Clinical Anatomy, Tbilisi State Medical University, Tbilisi 0186, Georgia; ^2^Department of Interventional Cardiology, NY Cardiovascular Research, New York, NY 10019, USA; ^3^Department of Child and Adolescent Maxillofacial Surgery and Surgical Stomatology, Tbilisi State Medical University, 0186 Tbilisi, Georgia

## Abstract

The acute respiratory distress syndrome (ARDS) is one of the main causes of high mortality in patients with coronavirus (COVID-19). In recent years, due to the coronavirus pandemic, the number of patients with ARDS has increased significantly. Unfortunately, until now, there are no effective treatments for ARDS caused by COVID-19. Many drugs are either ineffective or have a low effect. Currently, there have been reports of efficient use of mesenchymal stem cells (MSCs) for the treatment of ARDS caused by COVID-19. We investigated the influence of freeze-dried human placenta-derived mesenchymal stem cells (HPMSCs) in ARDS rat model. All animals have received intratracheal injection of 6 mg/kg of lipopolysaccharide (LPS). The rats were randomly divided into five groups: I: LPS, II: LPS+dexamethasone, III: LPS+HPMSCs, IV: HPMSC, and V: saline. ARDS observation time was short-term and amounted to 168 hours. The study has shown that HPMSCs are able to migrate and attach to damaged lung tissue, contributing to the resolution of pathology, restoration of function, and tissue repair in the alveolar space. Studies have also shown that the administration of HPMSCs in animals with ARDS model significantly reduced the levels of key cytokines such as IL-1*β*, IL-6, and TNF-*α*. Freeze-dried placental stem cell is a very promising biomaterial for the treatment of ARDS. The human placenta can be easily obtained because it is considered as a medical waste. At the same time, a huge number of MSCs can be obtained from the placental tissue, and there is no ethical controversy around their use. The freeze-dried MSCs from human placental tissue can be stored sterile at room temperature for a long time before use.

## 1. Introduction

Acute respiratory distress syndrome (ARDS) can cause severe lung damage. The overall mortality rate for patients with ARDS is approximately 35-40% [1]. Unfortunately, in recent years, due to coronavirus disease (COVID-19), the number of patients with ARDS has increased dramatically. The authors report that the mortality rate from ARDS in COVID-19 patients on mechanical ventilation ranges from 65.7% to 94% [2, 3]. The lack of a clear understanding of the biology and pathophysiology of the SARS-CoV-2 virus creates great problems in the search for effective treatment [4, 5].

In recent years, scientists around the world have been actively developing and researching for various potentially effective drugs for the treatment of the COVID-19. However, until this date, there is no antiviral treatment with confirmed effectiveness for COVID-19 [6]. Many drugs are either ineffective or have low effect. Others, on the other hand, have serious side effects.

Currently, interest is drawn towards the use of bone marrow stem cells (BMSCs) for the treatment of ARDS in COVID-19 patients. It has been reported that in animals with an ARDS model, administration of mesenchymal stem cells (MSCs) results in improved lung function [7]. It is noted that intratracheal or intravenous administration of MSCs mitigates inflammation by reducing levels of interleukin- (IL-) 1-*α*, IL-1*β*, IL-6, IL-8, IFN-*γ*, macrophage inflammatory protein- (MIP-) 1, MIP-2, and tumor necrosis factor- (TNF-) *α*. At the same time, as noted by authors, there was a simultaneous increase in the levels of IL-1 receptor antagonist (IL-1RN), IL-10, prostaglandin E2 (PGE2), lipoxin A4 (LXA4), and TNF-inducible gene- (TSG-) 6 [7].

The authors also reported that MSC transplantation in ARDS can reduce the number of apoptotic cells in the lungs and distal organs [8, 9] and protect alveolar macrophages from apoptosis induced by endotoxins in part by inhibiting the Wnt/*β*-catenin pathway [10]. Besides, MSC therapy can reduce TNF-*α* level [11]. Besides, MSC therapy can reduce TNF-*α* level [11]. It was found that cells represent sources of paracrine factors [12, 13]. MSCs are also able to secrete factors that enhance angiogenesis, for example, a factor that stimulates the colony of granulocytes, vascular endothelial growth factor (VEGF), hepatocyte growth factor, IL-6, chemotactic protein of monocytes-1, and TGF*β*1. [14, 15]. Additionally, there are reports that MSC paracrine factors stimulate the regeneration of damaged tissues, angiogenesis, and regulation of specific metabolic signaling pathways [16, 17]. There are suggestions that MSCs are capable of modulating macrophages, dendritic cells, neutrophils, natural killer cells, alveolar epithelial cells, and T and B-lymphocytes [18].

We hypothesized that freeze-dried MSCs derived from human placenta could be used to treat ARDS. We used freeze-dried MSCs derived from human placenta for the treatment of lipopolysaccharide-induced ARDS in rats.

## 2. Materials and Methods

### 2.1. Human Placenta Collection and HPMSC Isolation

Following written consent, placentas were collected from four women donors immediately after elective caesarean section.  Table 1 shows maternal age, gestational age at delivery, fetal and placental weights at delivery, fetal sex, and mode of delivery.

The newly acquired placentas were washed with 0.9% saline solution and transferred for processing to the laboratory within 10 minutes. To remove blood products, the placenta was washed using a polyethylene catheter inserted into the umbilical artery with 0.9% saline containing 100 U/ml penicillin and 100 *μ*g/ml streptomycin (Sigma-Aldrich). Perfusion was at a constant pressure of 5 ml/min using a peristaltic pump (Thermo Fisher Scientific). Perfusion was at a constant pressure of 5 ml/min using a peristaltic pump (Thermo Fisher Scientific). After perfusion and removal of decidua and fetal membranes, approximately 30-50 g of placental tissue was minced and washed in physiological saline (Oleg V Semenov et al.) [19]. Two protocols were used to isolate human placental MSCs: in the first protocol, placental mesenchymal stem cells were isolated by density gradient centrifugation using Ficoll Paque Plus (GE Healthcare Bio-Sciences, Pittsburgh, PA, USA) exactly as we described previously [13]. In the second protocol, the minced placental tissue was incubated in 50 mL 0.25% trypsin solution containing 80 U/mL DNase I for 1 h at 37°C. Cell suspensions were filtered twice through 150 *μ*m cell strainers (BD, Franklin Lakes, NJ, USA), and then, the cells were collected by centrifugation at the rate of 100 g for 5 minutes followed by washing with DMEM with 10% FBS two times. Before lyophilization, human placental MSCs were cultured in a culture medium (DMEM) and 20% fetal bovine serum (FBS) as a basal medium with addition 100 mg/mL streptomycin and 100 U/mL penicillin. The cells were cultured at 37°C in a 5% CO2 incubator and were identified based on their phenotypic characterization, which was performed after passage three. The placental stem cells were frozen (-20^о^C) and then lyophilized with the lyophilizer (HetoPower Dry PL6000 freeze drier; Sjia Lab, Shenzhen, China). First, the temperature of the lyophilizer shelf was set to -32°C and the vacuum at 10 Pa. The drying process lasted for 16 h. Dried stem cells were stored under sterile conditions at a room temperature until further use.

### 2.2. Flow Cytometry

To determine the expression of cell surface markers, cells were incubated with MSC-specific monoclonal antibodies such as CD31, CD34, CD45, CD73, CD90, CD105, CD133, and HLA-DR. All monoclonal antibodies (Miltenyi Biotec, Germany) were assessed according to the manufacturer's instructions. Stained cells were resuspended in PBS, analyzed using a FACS Calibur flow cytometer (Becton Dickinson). The calculated data were analyzed using the Cell Quest Pro software provided by the manufacturer.

### 2.3. Labeling and Tracking of the HPMSCs

HPMSCs were labeled with PKH67 Green fluorescent Cell Linker mini kit (Lot # MKC H8480; Sigma-Aldrich, MO, USA) by following the manufacturer's protocol. Freeze-dried HPMSCs were rehydrated in PBS and resuspended in Diluent C. Three *μ*L of PKH67 dye was added and incubated for 4 min at room temperature. Two milliliters of 1% BSA/PBS was added to bind excess dye. Afterwards, PKH26-labeled cells were centrifuged, lyophilized, and stored in sterile conditions until use.

### 2.4. Animals and Experimental Design

A total of 100 male outbred albino Wistar rats (aged 8 weeks, weighting ~250 g) were used for establishing a model of ARDS. The animals were acquired from the vivarium of Tbilisi State Medical University (Tbilisi, Georgia). The rats were maintained under controlled conditions at 24 ± 2°C using a 12 h light-dark cycle with provision of pelleted rodent diet and water ad libitum. All animals received care according to institutional guidelines.

We used LPS for creating a model of ARDS. Notably, LPS is widely used for studying ARDS in animals [20, 21].

The rats were randomly divided into five groups. All procedures were performed under anesthesia (70 mg/kg ketamine and 9 mg/kg xylazine; Sigma-Aldrich). After anesthesia, a 20 Fr catheter has been introduced into the trachea through the mouth in all animals, which then was connected to the artificial lung ventilation device. After 3 minutes, the device was turned off and LPS (from Escherichia coli K-235, product number: L2018, Sigma-Aldrich) dissolved in physiological solution was introduced through the catheter. Animals of the third group (*n* = 20), after LPS administration, were additionally HPMSC-labeled green fluorescent PKH67 was introduced through the catheter. The animals of the fourth group (*n* = 20) were intratracheally injected with HPMSC-labeled green fluorescent PKH67. The animals of the fifth (*n* = 20) group were intratracheally injected with saline. Afterwards, the rats were mechanically ventilated for 5 minutes. After the restoration of normal respiration, the animals were placed in a warm chamber for two hours and only after that, the animals were placed in standard laboratory conditions. The animals of the first group were observed without treatment. Animals of the second group received dexamethasone, which was injected intraperitoneally with the dose of 2.5 mg/kg. Injections were made daily throughout the observation period.

The rats were euthanized at 12, 24, 48, 96, 120, and 168 hours, using a combination of intraperitoneal injection of ketamine and xylazine, with subsequent laparotomy, aorta rupture, and exsanguinations.

### 2.5. Determination of the Levels of Cytokines

The blood samples of each rat were collected by retroorbital puncture using blood capillary tubes. Serum was obtained immediately by centrifugation of blood samples at 3000 g for 10 minutes. The expressions of IL-1*β* (IL-1 beta Rat ELISA Kit (Catalog #BMS630TEN, Invitrogen), IL-6 (IL-6 Rat ELISA Kit (Catalog #BMS625, Invitrogen), and TNF-*α* (TNF alpha Rat ELISA Kit, (Catalog #BMS622, Invitrogen) were analyzed by enzyme-linked immunosorbent assay (ELISA) technique (Invitrogen, Thermo Fisher Scientific) according to the manufacturer's instructions.

### 2.6. Wet-to-Dry Lung Weight Ratio

The wet-to-dry lung weight (W/D) ratios were used as an indicator of liquid accumulation in the lungs after the induction of LPS. The right lung was weighed immediately after its removal and afterwards, and it was dried for 5 minutes under the low power microwaves (200 W) to measure the dry weight. The W/D ratio was calculated as follows: *W*/*D* = wet weight/dry weight.

### 2.7. Histopathological Examination

For the histopathological examination, after the extraction of the left lung, it was immediately fixed in 10% neutral buffered formalin, embedded in paraffin, and cut into 5 *μ*m thick slices. The right lung was obtained to determine the wet-to-dry lung weight ratio. The lung tissues were stained with hematoxylin and eosin and Masson's trichrome according to the manufacturers' protocols. Immunostaining with anti-*α*-SMA antibodies (Abcam) was performed with the Novolink DAB Polymer Detection system (Leica Biosystems Newcastle Ltd) according to the manufacturer's recommendations. Endogenous peroxidase activity was neutralized using the Peroxidase Block reagent (hydrogen peroxide; Novolink DAB Polymer Detection system; Leica Biosystems Newcastle Ltd). Rabbit anti-mouse IgG was used as a secondary antibody (Novolink DAB Polymer Detection system).

### 2.8. Statistical Analysis

The GraphPad Prism 9.0 software (GraphPad Software, Inc.) was used to process statistical data. At each time point, the cytokine levels were estimated and compared among the groups. To compare the differences across the multiple groups, Tukey's post hoc tests and one-way variance analysis were used. All experiments were repeated at least three times. *P* < 0.05 was considered to indicate a statistically significant difference.

## 3. Results and Discussion

**Figure 1:**
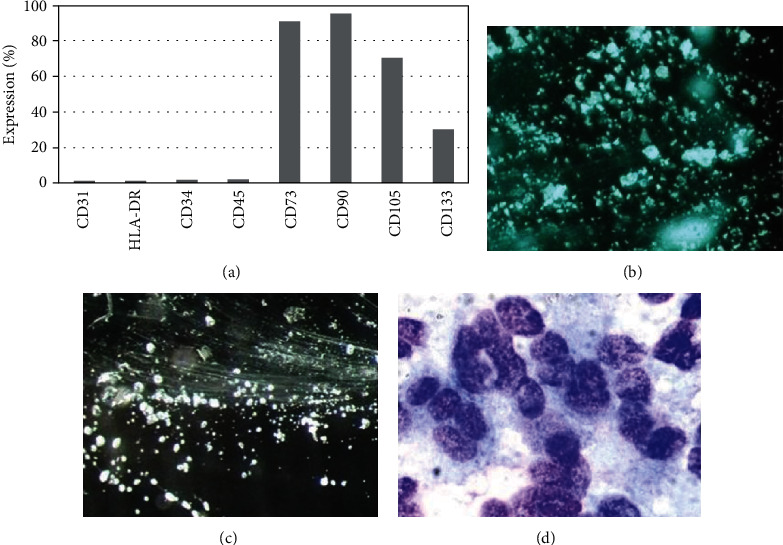
Immunophenotypic analysis of HPMSCs. (a) After isolation of human placental stem cells, immunophenotypical analysis was conducted with flow cytometry; (b) freeze-dried HPMSCs with stereoscopic microscopy (×20); (c) freeze-dried HPMSCs after rehydration with stereoscopic microscopy (×12); (d) Giemsa stain (×1000) of freeze-dried HPMSCs after rehydration.

Flow cytometry results showed that cell surface markers such as CD73, CD90, and CD105 were highly expressed, while CD31, CD34, HLA-DR, and CD45 showed low expression, consistent with MSC profiles. We hypothesized that cells isolated from placental tissue may have characteristics of MSCs (Figure 1).

**Figure 2:**
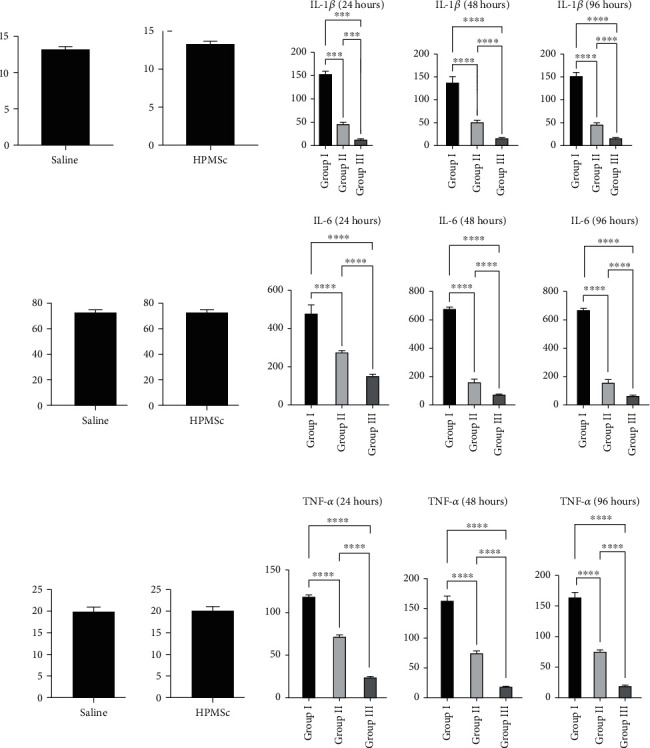
HPMSCs and analysis of cytokine levels after modeling and treatment of ARDS. Analysis of cytokine levels. Intratracheal injection of LPS increased expression levels the proinflammatory cytokine. In animals of the first and second groups, during the first three days, the levels of inflammatory cytokines TNF-*α*, IL-1*β*, and IL-6 in the blood serum were significantly increased and remained at the same level during the entire observation period. The introduction of HPMSCs significantly limited the increase of inflammation marker levels in the third group (^∗∗∗∗^*P* < 0.0001).

Intratracheal injection of LPS increased expression levels of the proinflammatory cytokine. In animals of the first and second groups, during the first three days, the levels of inflammatory cytokines TNF-*α*, IL-1*β*, and IL-6 in the blood serum were significantly increased and remained at the same level during the entire period of observation of the animals. The introduction of HPMSCs significantly limited the increase of inflammation marker levels in the third group (Figure 2).

At autopsy, the lungs of animals of the first (LPS) and second (LPS+dexamethasone) groups were hyperemic and edematous. Purple spots and hemorrhages were visible on the surface of the lungs. The lungs of the animals of the third group (LPS+HPMSCs) were slightly hyperemic. On the surface of the lungs, purple spots and hemorrhages were not detected. The lungs of the animals of the fourth (HPMSCs) and fifth (Saline) groups did not differ from the lungs of normal animals.

Compared to the animals of the second and third groups, the *W*/*D* ratio was significant in the first group. The only difference was that the *W*/*D* ratio has returned to the initial level after 96 hours in the animals of the third group.

**Figure 3:**
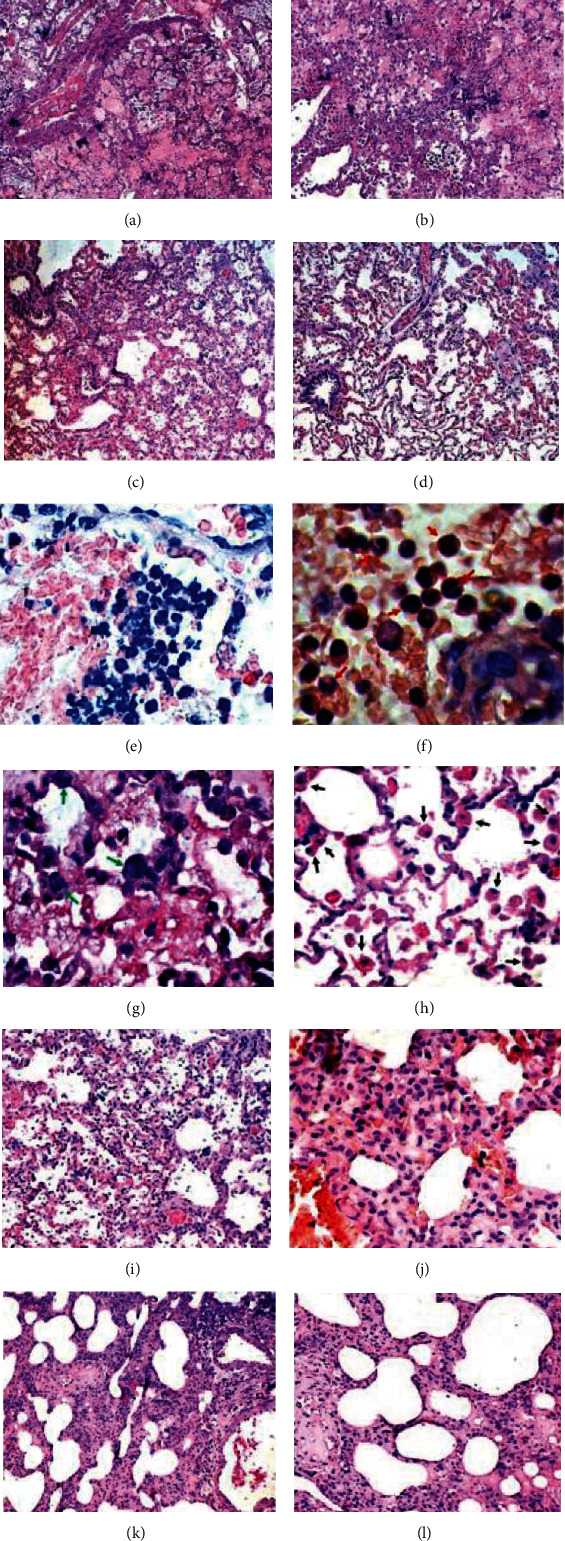
Histological changes in lung tissue after ARDS modeling and treatment. (a) LPS and the (b) LPS+dexamethasone groups. Structural lung damage, edema, alveolar hemorrhage, and (e) inflammatory cell infiltration. H&E staining ×200. Observation period 48 hours; (c) LPS+HPMSC and (d) HPMSC groups. The mild edema and infiltration of the lung tissue with inflammatory cells. Observation period 48 hours. H&E staining ×200; (f) LPS and the LPS+dexamethasone groups. Neutrophils (red arrows), (g) giant cells (green arrows), and (h) a large number of alveolar macrophages were noted in the alveolar space. Observation period 96 hours. H&E staining ×800; (i) LPS group. Destruction of lung tissue. H&E staining ×200. (j) LPS+dexamethasone group. Thickening of the alveolar septa. H&E staining ×400; (k) LPS+HPMSC group. A slight thickening of the alveolar septum. H&E staining ×200; (l) the HPMSC group. A solid structure, clear alveolar space without congestion in the alveolar wall. H&E staining ×200. Observation period one week.

**Figure 4:**
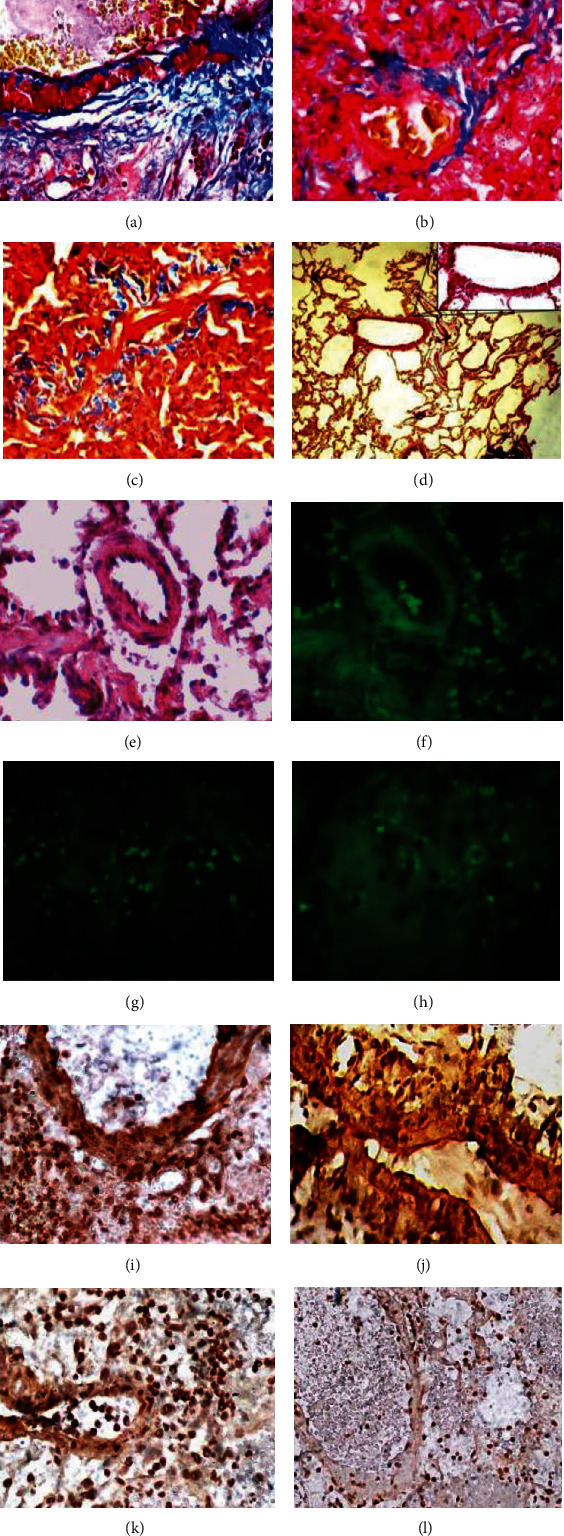
Masson's trichrome staining, immunohistochemistry, and the fluorescence microscopy of the lung section after ARDS modeling and treatment. (a, b) LPS and LPS+dexamethasone groups. The intense blue staining of collagen fibers surrounding the bronchi and alveolar vessels. (c) Blue staining of collagen fibers in the lung interstitium. Masson's trichrome staining ×800. (d) LPS+HPMSC group. Slight blue coloration of collagen fibers around the vessels. Masson's trichrome staining ×200/800. Observation period one week. (e–h) LPS+HPMSC group. HPMSCSs labeled with the PKH26 fluorescent dye on the sections of lung tissue. (e) At 24 hours after their intratracheal injection; H&E staining ×800. (f) Fluorescence microscopy; observation period 24 hours. Magnification ×800; (g, h) Fluorescence microscopy. Observation period 96 hours. Magnification ×800; (i, j) LPS group. *α*-SMA-positive cells appeared in the area of fibrotic lesions. Magnification ×400/800 (dark brown staining); (k) LPS+dexamethasone group. Intensive staining of *α*-SMA-positive cells. Magnification ×400; (l) LPS+HPMSC group. Weak *α*-SMA immunostaining signal. Magnification ×400. Observation period one week.

At 48 hours, in the LPS and the LPS+dexamethasone groups, structural damage, edema, and alveolar hemorrhage were evident. H&E staining of the lung section also showed alveoli filled with pink protein aceous material (Figures 3(a) and 3(b)). The infiltration of lung tissue with inflammatory cells was obvious (Figure 3(e)). A large number of neutrophils were noted in the alveolar space (Figure 3(f)). Structural damage in the LPS+HPMSC group was significantly reduced. Only mild edema and infiltration of the lung tissue with inflammatory cells can be seen (Figure 3(c)). In animals of the fourth group, insignificant infiltration of lung tissue with inflammatory cells was observed (Figure 3(d)). After 96 hours (LPS group and LPS+dexamethasone group), giant cells (Figure 3(g)) and a large number of alveolar macrophages were noted in the alveolar space (Figure 3(h)). Destruction of lung tissue was noted in the LPS group after 168 hours (Figure 3(i)). At the same time, thickening of the alveolar septa was noted in the LPS+dexamethasone group (Figure 3(j)). A mild thickening of the alveolar septum was noted in the LPS+HPMSC group (Figure 3(k)). Opposite, a solid structure, clear alveolar space without congestion in the alveolar wall was displayed in the HPMSC group (Figure 3(l)). A week after, in the LPS and LPS+dexamethasone groups, Masson's trichrome staining showed the presence of fibrosis, which was expressed as an intense blue staining of collagen fibers surrounding the vessels and bronchioles (Figure 4(a)). Blue coloration of collagen fibers was also noted around the alveolar vessels and in the interstitium (Figures 4(b) and 4(c)). This result was not observed in the LPS+HPMSC group (Figure 4(d)). After 24 hours, fluorescent PKH26-labeled HPMSCs were present on the lung tissue sections (Figures 4(e) and 4(f)). HPMSCs labeled with PKH26 were also detected after 48 hours; however, after 96 hours, the amount of HPMSCs labeled with PKH26 fluorescent dye was significantly reduced (Figures 4(g) and 4(h)). In the LPS and LPS+dexamethasone groups, *α*-SMA-positive cells appeared in the area of fibrotic lesions 96 hours after modeling (Figures 4(i)–4(k)). In the LPS+HPMSC group, at the same time, a very weak *α*-SMA immunostaining signal was observed (Figure 4(l)).

In this study, we investigated the efficiency of freeze-dried human placental mesenchymal stem cells in an LPS-induced ARDS rat model. Despite decades of research, the prospects for effective treatment for ARDS remain bleak. A particularly difficult situation has developed in the treatment of COVID-19 patients. As reported by many authors, clinical trials using corticosteroids, prostaglandins, nitric oxide, prostacyclin, surfactant, lysophylline, ketoconazole, and N-acetylcysteine failed to show a statistically significant improvement in patient mortality [22]. Surfactant therapy is not always effective. A Cochrane review does not recommend its use in adults [23]. The use of corticosteroids for ARDS is controversial [24]. Currently, various drugs are being actively developed for the effective treatment of ARDS caused by COVID-19 virus, including antiviral drugs (lopinavir, remdesivir, favipiravir, etc.), antiparasitic drugs (hydroxychloroquine, nitazoxanide, etc.), and corticosteroids, monoclonal antibodies (lenzilumab, etc.) as well as transfusion of convalescent plasma.

Most of these drugs have shown clear improvements in animal survival in preclinical studies but have failed to show similar results in humans [22, 25]. Currently, cell therapy is considered a promising treatment strategy for ARDS. Preclinical studies in animals with induced lung injury have shown that administration of MSCs can reduce inflammation and limit lung damage and significantly reduce mortality [26, 27]. There are reports that intrapulmonary delivery of bone marrow mesenchymal stem cells increases survival in mice with an acute lung injury model [28]. MSCs with antimicrobial activity can be effectively used in the treatment of pulmonary infections [29]. The authors note that MSCs secrete the antimicrobial peptide LL-37, exerting an antimicrobial effect and stimulating bacterial clearance [30, 31].

There is an interesting report on the effectiveness of MSCs in the treatment of sepsis-induced ARDS. As it is known, sepsis is a complex clinical syndrome with physiological, biological, and biochemical abnormalities [32]. The lungs are reported to be the most sepsis-prone organ in which damage to alveolar type II epithelial cells and capillary endothelial cell (EC) can cause ARDS [33, 34]. In this study, the authors examined the molecular defense mechanisms provided by MSCs in sepsis. They identified three general effects of MSC administration: (a) weakening of sepsis-induced functional impairment of mitochondria, (b) suppression of proinflammatory transcriptional responses of endotoxin/toll-like innate immunity receptor and proinflammatory transcriptional responses, and (c) coordinated expression of transcriptional programs involved in maintaining the integrity of the vascular endothelium [35]. To date, preclinical and clinical studies have confirmed the therapeutic effect of MSCs in ARDS, including those caused by the COVID-19 virus [36–43].

It is important to note that the anti-inflammatory effects of MSCs are mainly explained by paracrine mechanisms, since, as the authors have reported, after MSC therapy, few or no transplanted donor stem cells are localized in the host's lung tissue [44].

Moreover, MSCs protect the endothelial barrier complex and survival involved in the pathogenesis of acute lung injury (ALI) via paracrine hepatocyte growth factor (HGF). As reported by the authors, the activation of the mTOR/STAT-3 pathway provides novel mechanistic insights into MSC-secreted HGF protection against LPS-induced vascular endothelial permeability dysfunction and apoptosis, which contributes to decreasing microvascular loss and lung injury [45, 46].

We hypothesized that freeze-dried HPMSCs could be used to treat ARDS especially in COVID-19 patients. For determining the effectiveness of freeze-dried HPMSCs, we created an ARDS model in rats using LPS. It is accepted that an ideal animal model of ARDS must match the characteristics of human ARDS that include rapid onset, development of physiological dysfunction, and damage to the lung parenchyma; however, as the authors note, not all animal models are likely to have all major features of ARDS observed in humans [47]. It is reported that the most practical and invariable small animal model of lung injury is acquired through the administration of LPS [48].

In this article, we will not describe the advantages and disadvantages of the LPS model in animals. Many authors [49, 50] have already described them. We are only underlining the fact that after intratracheal administration of LPS, the ARDS was evident in all animal groups, which was characterized by a rapid onset and lung damage.

At autopsy, the lungs of animals were hyperemic and edematous. Purple spots and hemorrhages were visible on the surface of the lungs. After the ARDS modeling, the histological examinations have shown the thickening of interalveolar septa, edema, and extensive infiltration of inflammatory cells. With the Masson's trichrome staining, deposition of collagen was noted in the wall of the bronchi and interstitium. Immunohistochemical methods of studying animals showed extensive expression of actin-positive cells in the wall of the bronchioles and adjacent blood vessels. Thus, we confirm that the most practical small animal model of lung injury is acquired through the administration of LPS.

Our attention was drawn to the placenta, which until this date still is considered as the mysterious “least understood organ” [51, 52]. In ancient times, the placenta was considered animistic, possessing mind or spirit. [53]. For decades, clinicians have been using placental tissue fragments, amniotic and chorionic membranes, umbilical cord, placental extracts, and lyophilisates.

Placenta offers a rich source of stem cells that include trophoblastic, haematopoietic, epithelial, and MSCs [54, 55]. According to the reports, amniotic epithelium cells from term placenta express several stem cell surface markers that are commonly found on pluripotent stem cells such as embryonic stem cells [56]. There are reports that stem cells have been found in the amniotic fluid and placenta, which sustained long-term undifferentiated proliferation and differentiated into several tissue types spanning the three germ layers [57].

There are examples effective application of placenta tissue in abdominal and pelvic surgeries [58, 59], cardiac surgery [60, 61], for wound healing [62, 63], for treatment of acute chemical or thermal burns [64], in ophthalmology, for ocular surface reconstruction, including the treatment of persistent epithelial defects and nonhealing corneal ulcers [65], and etc.

Our interest was aroused by articles related to the use of placental tissue for abrogates lung fibrosis. The authors also note that human amnion epithelial cell administration reduces inflammation in association with decreased monocyte chemoattractant protein-1, tumor necrosis factor-alpha, IL-1 and IL-6, and profibrotic transforming growth factor-beta in the lungs of mice. The authors noted that the lung collagen content was significantly reduced by hAEC treatment as a possible consequence of increased degradation by matrix metalloproteinase-2 and downregulation of the tissue inhibitors of matrix metalloproteinase-1 and 2 [66]. Similar studies have been demonstrated by other authors stating that human amnion epithelial cells prevent bleomycin-induced lung injury and preserve lung function [67, 68].

The authors report that under standard cryopreservation procedures, cell recovery rates vary from 87.67% to 94.76% [69, 70]. According to the studies, without addition of protectors, lyophilization ensures up to 70% viability of MSCs [71, 72].

In our studies, the percentage of viable fresh cells before the procedure was 92%, after cryopreservation and thawing, the percentage of viable cells was 82%, and the viability of cells after freeze-drying and rehydration was 53%. Cell viability was determined by trypan blue and standard light microscopy. Freeze-drying of MSCs of the placenta was performed without the addition of protectors.

It should be noted that for many years, various methods have been developed that increase the resistance of cells to desiccation. It is known that the presence of trehalose on both sides of the cell membrane increases the resistance of mammalian cells to desiccation. However, trehalose is impermeable to the cell membrane, which significantly limits the possibilities of its application. To solve this problem, the authors have used a high-capacity trehalose transporter (TRET1) from the African chironomid Polypedilum vanderplanki to introduce trehalose into the cytoplasm of mammalian cells [73]. The authors note that after desiccation to 2.60 g of water per gram dry weight, in comparison with the control CHO cells, a 170% increase in viability and a 400% increase in growth (after 7 days) was observed for CHO-TRET1. However, the problem of creating effective protectors for adequate protection of intracellular components and the cell membrane of MSCs during drying still remains. Based on the abovementioned, our main focus was on the growth factors that are present in lyophilized placental MSCs and their role in stimulating the regeneration of damaged lungs.

Our data confirms the ability of HPMSCs to migrate and attach to damaged lung tissue, contributing to the resolution of pathology, restoration of function, and tissue repair in the alveolar space. These HPMSC effects appear to be mediated by paracrine factors, although, after 96 hours, the amount of HPMSCs labeled with PKH26 fluorescent dye was significantly reduced. Other authors have also reported that the anti-inflammatory effects of MSCs have been mostly attributed to paracrine/endocrine mechanisms [7].

It is reported that after lyophilization BMSC is observed to retain >80% of paracrine factors, including VEGF-1, insulin-like growth factor 1, EGF, hepatocyte growth factor, keratinocyte growth factor, angiopoietin 1, factor-1 derived from stromal cells, chemoattractant protein of monocyte-1, and erythropoietin [74, 75]. It is also reported that resolution of ARDS can be improved by the release of several paracrine factors produced by MSCs, which restore lung function [76].

Our earlier studies have shown that the decellularized and lyophilized human placenta tissue contains numerous growth factors such as EGF, bFGF, KGF, VEGF, TGF-a, TGF-b, PDGF, HGF, and NGF [13]. Thus, based on our own research and analysis of the literature, we can assume that freeze-dried stem cells of the human placenta, as well as decellularized tissue of the human placenta, have antimicrobial, anti-inflammatory, and immunomodulatory properties [77–80].

The results of our research have demonstrated that in comparison with the animals that received no treatment or received only dexamethasone, the administration of HPMSCs in the animals with ARDS model significantly reduced the levels of key cytokines such as IL-1*β*, IL-6, and TNF-*α*. Our results are consistent with those of other authors who report that MSCs decrease the expression of several proinflammatory cytokines such as TNF-*α*, IL-1*β*, IL-6, and IFN-*γ* and increase anti-inflammatory cytokines such as IL-4 and IL-10 [81].

The role of the cytokine storm in the development of ARDS in COVID-19 patients is being actively discussed in the literature [82]. Cytokine storms can occur with viral infections such as H1N1 influenza, H5N1 influenza [83, 84], and SARS-CoV-2 [85]. As reported by many authors, the inflammatory process caused by viral infection induces the stimulation of proinflammatory cytokines such as IL-1*β*, IL-2, IL-6, IL-7, IL-8, IL-10, IFN-*γ*, and TNF-*α* [86, 87].

The prognosis of COVID-19 may worsen significantly due to overproduction of mainly proinflammatory cytokines such as IL-1, IL-6, IL-12, IFN-*γ*, and TNF-*α*, which mainly target the lung tissue [88]. The role of multiple immunological processes that engage neutrophils, macrophages, and dendritic cells involved in lung tissue damage in ARDS should also be noted [89].

Based on this, result can be said that HPMSCs are safe and effective for ARDS treatment. However, we believe that for the successful use of HPMSCs in clinical practice, it is necessary to understand better their role in the mechanism of repair of the lung damaged.

## 4. Conclusion

Our research confirms that HPMSCs have ability to migrate and attach to damaged lung tissue, contributing to the resolution of pathology, restoration of function, and tissue repair in the alveolar space. Freeze-dried placental stem cell is a very promising biomaterial that it can be used for the treatment of ARDS, especially for cases caused by COVID-19. The human placenta can be obtained because it is considered as a medical waste. At the same time, a huge number of MSCs can be obtained from the placental tissue, and there is no ethical controversy regarding its use. The freeze-dried MSCs from human placental tissue can be stored in sterile conditions at a room temperature for a long time until use. However, in order to use HPMSCs successfully, it is first necessary to understand the mechanism of damaged lung repair in ARDS using HPMSC paracrine factors. It is also necessary to resolve issues such as optimal timing and duration of administration, dose, and optimal delivery route. This requires more extensive and thorough research.

## Figures and Tables

**Table 1:** Maternal age, gestational age at delivery, fetal and placental weights at delivery, fetal sex, and mode of delivery.

Human placentas	Maternal age	Gestational age at delivery	Fetal weight at delivery (g)	Placental weight at delivery (g)	Fetal sex	Mode of delivery
1	28 y/o	38 weeks	3382	564	Feminine	Caesarean section
2	32 y/o	40 weeks	3580	597	Feminine	Caesarean section
3	35 y/o	39 weeks	3600	600	Feminine	Caesarean section
4	41 y/o	40 weeks	3600	600	Masculine	Caesarean section

## Data Availability

All data are included in the article.

## Abbreviations

BM-MSCs: Bone marrow-derived mesenchymal stem cells
PF: Paracrine factors
HPMSCs: Human placental mesenchymal stem cells
ARDS: Acute respiratory distress syndrome
MIP: Macrophage inflammatory protein
PBS: Phosphate-buffered saline
TNF-*α*: Tumor necrosis factor-*α*
IL: Interleukin
IL-1RN: IL-1 receptor antagonist
PGE2: Prostaglandin E2
LXA4: Lipoxin A4
LPS: Lipopolysaccharides
TSG: TNF-inducible gene
VEGF: Vascular endothelial growth factor
FBS: Fetal bovine serum
DMEM: Dulbecco's modified Eagle's medium
ANOVA: Analysis of variance.

## References

[1] Bellani G., Laffey J. G., Pham T. (2016). Epidemiology, patterns of care, and mortality for patients with acute respiratory distress syndrome in intensive care units in 50 countries. *JAMA*.

[2] Gibson P. G., Qin L., Puah S. H. (2020). COVID-19 acute respiratory distress syndrome (ARDS): clinical features and differences from typical pre-COVID-19 ARDS. *The Medical Journal of Australia*.

[3] Wu C., Chen X., Cai Y. (2020). Risk factors associated with acute respiratory distress syndrome and death in patients with coronavirus disease 2019 pneumonia in Wuhan, China. *JAMA Internal Medicine*.

[4] Feng W., Zong W., Wang F., Ju S. (2020). Severe acute respiratory syndrome coronavirus 2 (SARS-CoV-2): a review. *Molecular Cancer*.

[5] Sridhar S., Nicholls J. (2021). Pathophysiology of infection with SARS-CoV-2-what is known and what remains a mystery. *Respirology*.

[6] Li L., Li R., Wu Z. (2020). Therapeutic strategies for critically ill patients with COVID-19. *Annals of Intensive Care*.

[7] Lopes-Pacheco M., Robba C., Rocco P. R. M., Pelosi P. (2020). Current understanding of the therapeutic benefits of mesenchymal stem cells in acute respiratory distress syndrome. *Cell Biology and Toxicology*.

[8] Bingyu X., Lu C., Wang X., Yongjia Z., Wang Y., Charlie X. (2017). Transplantation of menstrual blood-derived mesenchymal stem cells promotes the repair of LPS-induced acute lung injury. *International Journal of Molecular Sciences*.

[9] Silva J. D., Lopes-Pacheco M., Paz A. H. R. (2018). Mesenchymal stem cells from bone marrow, adipose tissue, and lung tissue differentially mitigate lung and distal organ damage in experimental acute respiratory distress syndrome. *Critical Care Medicine*.

[10] Li B., Zhang H., Zeng M. (2015). Bone marrow mesenchymal stem cells protect alveolar macrophages from lipopolysaccharide-induced apoptosis partially by inhibiting the Wnt/*β*-catenin pathway. *Cell Biology International*.

[11] Kim E. S., Chang Y. S., Choi S. J. (2011). Intratracheal transplantation of human umbilical cord blood-derived mesenchymal stem cells attenuates Escherichia coli-induced acute lung injury in mice. *Respiratory Research*.

[12] Liang X., Ding Y., Zhang Y., Tse H., Lian Q. (2014). Paracrine mechanisms of mesenchymal stem cell-based therapy: current status and perspectives. *Cell Transplantation*.

[13] Kakabadze Z., Chakhunashvili D., Gogilashvili K. (2019). Bone marrow stem cell and decellularized human amniotic membrane for the treatment of nonhealing wound after radiation therapy. *Experimental and Clinical Transplantation*.

[14] Kwon H. M., Hur S. M., Park K. Y. (2014). Multiple paracrine factors secreted by mesenchymal stem cells contribute to angiogenesis. *Vascular Pharmacology*.

[15] Baraniak P. R., McDevitt T. C. (2010). Stem cell paracrine actions and tissue regeneration. *Regenerative Medicine*.

[16] Hocking A. M., Gibran N. S. (2010). Mesenchymal stem cells: paracrine signaling and differentiation during cutaneous wound repair. *Experimental Cell Research*.

[17] Burdon T. J., Paul A., Noiseux N., Prakash S., Shum Tim D. (2011). Bone marrow stem cell derived paracrine factors for regenerative medicine: current perspectives and therapeutic potential. *Bone Marrow Research*.

[18] Guillamat-Prats R., Camprubí-Rimblas M., Bringué J., Tantinyà N., Artigas A. (2017). Cell therapy for the treatment of sepsis and acute respiratory distress syndrome. *Annals of Translational Medicine*.

[19] Semenov O. V., Koestenbauer S., Riegel M. (2010). Multipotent mesenchymal stem cells from human placenta: critical parameters for isolation and maintenance of stemness after isolation. *American Journal of Obstetrics and Gynecology*.

[20] Chimenti L., Morales-Quinteros L., Puig F. (2020). Comparison of direct and indirect models of early induced acute lung injury. *Intensive Care Medicine Experimental*.

[21] Domscheit H., Hegeman M. A., Carvalho N., Spieth P. M. (2020). Molecular dynamics of lipopolysaccharide-induced lung injury in rodents. *Frontiers in Physiology*.

[22] Jain R., DalNogare A. (2006). Pharmacological therapy for acute respiratory distress syndrome. *Mayo Clinic Proceedings*.

[23] Adhikari N., Burns K. E., Meade M. O. (2004). Pharmacologic therapies for adults with acute lung injury and acute respiratory distress syndrome. *Cochrane Database of Systematic Reviews*.

[24] Yang J. W., Jiang P., Wang W. W. (2021). The controversy about the effects of different doses of corticosteroid treatment on clinical outcomes for acute respiratory distress syndrome patients: an observational study. *Frontiers in Pharmacology*.

[25] Baron R. M., Levy B. D. (2016). Recent advances in understanding and treating ARDS. *F1000Research*.

[26] Matthay M. A., Goolaerts A., Howard J. P., Lee J. W. (2010). Mesenchymal stem cells for acute lung injury: preclinical evidence. *Critical Care Medicine*.

[27] McIntyre L. A., Moher D., Fergusson D. A. (2016). Canadian Critical Care Translational Biology Group. Efficacy of mesenchymal stromal cell therapy for acute lung injury in preclinical animal models: a systematic review. *PLoS One*.

[28] Gupta N., Su X., Popov B., Lee J. W., Serikov V., Matthay M. A. (2007). Intrapulmonary delivery of bone marrow-derived mesenchymal stem cells improves survival and attenuates endotoxin-induced acute lung injury in mice. *Journal of Immunology*.

[29] Li W., Chen W., Huang S., Tang X., Yao G., Sun L. (2020). Mesenchymal stem cells enhance pulmonary antimicrobial immunity and prevent following bacterial infection. *Stem Cells International*.

[30] Krasnodembskaya A., Song Y., Fang X. (2010). Antibacterial effect of human mesenchymal stem cells is mediated in part from secretion of the antimicrobial peptide LL-37. *Stem Cells*.

[31] Alcayaga-Miranda F., Cuenca J., Khoury M. (2017). Antimicrobial activity of mesenchymal stem cells: current status and new perspectives of antimicrobial peptide-based therapies. *Frontiers in Immunology*.

[32] Singer M., Deutschman C. S., Seymour C. W. (2016). The third international consensus definitions for sepsis and septic shock (sepsis-3). *Journal of the American Medical Association*.

[33] Hotchkiss R. S., Nicholson D. W. (2006). Apoptosis and caspases regulate death and inflammation in sepsis. *Nature Reviews. Immunology*.

[34] Thompson B. T., Chambers R. C., Liu K. D. (2017). Acute respiratory distress syndrome. *The New England Journal of Medicine*.

[35] dos Santos C. C., Murthy S., Hu P. (2012). Network analysis of transcriptional responses induced by mesenchymal stem cell treatment of experimental sepsis. *The American Journal of Pathology*.

[36] Harrell C. R., Jovicic B. P., Djonov V., Volarevic V. (2020). Therapeutic potential of mesenchymal stem cells and their secretome in the treatment of SARS-CoV-2-induced acute respiratory distress syndrome. *Analytical Cellular Pathology (Amsterdam)*.

[37] Can A., Coskun H. (2020). The rationale of using mesenchymal stem cells in patients with COVID-19-related acute respiratory distress syndrome: what to expect. *Stem Cells Translational Medicine*.

[38] Leng Z., Zhu R., Hou W. (2020). Transplantation of ACE2 (-) mesenchymal stem cells improves the outcome of patients with COVID-19 pneumonia. *Aging and Disease*.

[39] Sanchez-Guijo F., García-Arranz M., Lopez-Parra M. (2020). Adipose-derived mesenchymal stromal cells for the treatment of patients with severe SARS-CoV-2 pneumonia requiring mechanical ventilation. A proof of concept study. *EClinicalMedicine*.

[40] Wilson J. G., Liu K. D., Zhuo H. (2015). Mesenchymal stem (stromal) cells for treatment of ARDS: a phase 1 clinical trial. *The Lancet Respiratory Medicine*.

[41] Liu K. D., Wilson J. G., Zhuo H. (2014). Design and implementation of the START (STem cells for ARDS treatment) trial, a phase 1/2 trial of human mesenchymal stem/stromal cells for the treatment of moderate-severe acute respiratory distress syndrome. *Annals of Intensive Care*.

[42] Zheng G., Huang L., Tong H. (2014). Treatment of acute respiratory distress syndrome with allogeneic adipose-derived mesenchymal stem cells: a randomized, placebo-controlled pilot study. *Respiratory Research*.

[43] Sengupta V., Sengupta S., Lazo A., Woods P., Nolan A., Bremer N. (2020). Exosomes derived from bone marrow mesenchymal stem cells as treatment for severe COVID-19. *Stem Cells and Development*.

[44] Eggenhofer E., Benseler V., Kroemer A. (2012). Mesenchymal stem cells are short-lived and do not migrate beyond the lungs after intravenous infusion. *Frontiers in Immunology*.

[45] Meng S. S., Guo F. M., Zhang X. W. (2019). mTOR/STAT-3 pathway mediates mesenchymal stem cell-secreted hepatocyte growth factor protective effects against lipopolysaccharide-induced vascular endothelial barrier dysfunction and apoptosis. *Journal of Cellular Biochemistry*.

[46] Yang Y., Chen Q. H., Liu A. R., Xu X. P., Han J. B., Qiu H. B. (2015). Synergism of MSC-secreted HGF and VEGF in stabilising endothelial barrier function upon lipopolysaccharide stimulation via the Rac1 pathway. *Stem Cell Research & Therapy*.

[47] Matute-Bello G., Downey G., Moore B. B. (2011). Acute Lung Injury in Animals Study Group. An official American Thoracic Society workshop report: features and measurements of experimental acute lung injury in animals. *American Journal of Respiratory Cell and Molecular Biology*.

[48] Paris A. J., Guo L., Dai N. (2019). Using selective lung injury to improve murine models of spatially heterogeneous lung diseases. *PLoS One*.

[49] Matute-Bello G., Frevert C. W., Martin T. R. (2008). Animal models of acute lung injury. *American Journal of Physiology. Lung Cellular and Molecular Physiology*.

[50] Bastarache J. A., Blackwell T. S. (2009). Development of animal models for the acute respiratory distress syndrome. *Disease Models & Mechanisms*.

[51] Mir I. N., Chalak L. (2017). Placenta -‘the least understood human organ’- from animistic origins to human placental project. *Annals of Reproductive Medicine and Treatment*.

[52] Guttmacher A. E., Maddox Y. T., Spong C. Y. (2014). The human placenta project: placental structure, development, and function in real time. *Placenta*.

[53] Longo L. D., Reynolds L. P. (2010). Some historical aspects of understanding placental development, structure and function. *The International Journal of Developmental Biology*.

[54] Parolini O., Alviano F., Bagnara G. P. (2008). Concise review: isolation and characterization of cells from human term placenta: outcome of the first international workshop on placenta derived stem cells. *Stem Cells*.

[55] Yen B. L., Huang H.-I., Chien C.-C. (2005). Isolation of multipotent cells from human term placenta. *Stem Cells*.

[56] Miki T., Strom S. C. (2006). Amnion-derived pluripotent/multipotent stem cells. *Stem Cell Reviews*.

[57] Delo D. M., De Coppi P., Bartsch G., Atala A. (2006). Amniotic fluid and placental stem cells. *Methods in Enzymology*.

[58] Trelford-Sauder M., Dawe E. J., Trelford J. D. (1978). Use of allograft amniotic membrane for control of intra-abdominal adhesions. *Journal of Medicine*.

[59] Nassif J., Abbasi S. A., Kechli M. K. (2016). Effect of the mode of application of cryopreserved human amniotic membrane on adhesion formation after abdomino-pelvic surgery in a mouse model. *Frontiers in Medicine*.

[60] Marsh K. M., Ferng A. S., Pilikian T. (2017). Anti-inflammatory properties of amniotic membrane patch following pericardiectomy for constrictive pericarditis. *Journal of Cardiothoracic Surgery*.

[61] Peirovi H., Rezvani N., Hajinasrollah M., Mohammadi S. S., Niknejad H. (2012). Implantation of amniotic membrane as a vascular substitute in the external jugular vein of juvenile sheep. *Journal of Vascular Surgery*.

[62] ElHeneidy H., Omran E., Halwagy A., Al-Inany H., Al-Ansary M., Gad A. (2016). Amniotic membrane can be a valid source for wound healing. *International Journal of Women's Health*.

[63] Nouri M., Ebrahimi M., Bagheri T. (2018). Healing effects of dried and acellular human amniotic membrane and mepitelas for coverage of skin graft donor areas; a randomized clinical trial. *Bulletin of Emergency and Trauma*.

[64] Meller D., Pires R. T., Mack R. J. (2000). Amniotic membrane transplantation for acute chemical or thermal burns. *Ophthalmology*.

[65] Jirsova K., Jones G. L. A. (2017). Amniotic membrane in ophthalmology: properties, preparation, storage and indications for grafting-a review. *Cell and Tissue Banking*.

[66] Moodley Y., Ilancheran S., Samuel C. (2010). Human amnion epithelial cell transplantation abrogates lung fibrosis and augments repair. *American Journal of Respiratory and Critical Care Medicine*.

[67] Murphy S., Lim R., Dickinson H. (2011). Human amnion epithelial cells prevent bleomycin-induced lung injury and preserve lung function. *Cell Transplantation*.

[68] Cargnoni A., Ressel L., Rossi D. (2012). Conditioned medium from amniotic mesenchymal tissue cells reduces progression of bleomycin-induced lung fibrosis. *Cytotherapy*.

[69] Luo Y., Huang H., Yu J., Liang B. (2006). Effect of cryopreservation on the biological characteristics of adult mesenchymal stem cells derived from bone marrow. *Chinese Journal of Physiology*.

[70] Carvalho K. A., Cury C. C., Oliveira L. (2008). Evaluation of bone marrow mesenchymal stem cell standard cryopreservation procedure efficiency. *Transplantation Proceedings*.

[71] Bissoyi A., Kumar A., Rizvanov A. A. (2016). Recent advances and future direction in lyophilisation and desiccation of mesenchymal stem cells. *Stem Cells International*.

[72] Zhang S.-Z., Qian H., Wang Z. (2010). Preliminary study on the freeze-drying of human bone marrow-derived mesenchymal stem cells. *Journal of Zhejiang University: Science B*.

[73] Chakraborty N., Menze M. A., Elmoazzen H. (2012). Trehalose transporter from African chironomid larvae improves desiccation tolerance of Chinese hamster ovary cells. *Cryobiology*.

[74] Peng Y., Xuan M., Zou J. (2015). Freeze-dried rat bone marrow mesenchymal stem cell paracrine factors: a simplified novel material for skin wound therapy. *Tissue Engineering Part A*.

[75] Hsiao S. T., Asgari A., Lokmic Z. (2012). Comparative analysis of paracrine factor expression in human adult mesenchymal stem cells derived from bone marrow, adipose, and dermal tissue. *Stem Cells and Development*.

[76] Lee J. W., Fang X., Krasnodembskaya A., Howard J. P., Matthay M. A. (2011). Concise review: mesenchymal stem cells for acute lung injury: role of paracrine soluble factors. *Stem Cells*.

[77] Klama-Baryła A., Rojczyk E., Kitala D. (2020). Preparation of placental tissue transplants and their application in skin wound healing and chosen skin bullous diseases - Stevens-Johnson syndrome and toxic epidermal necrolysis treatment. *International Wound Journal*.

[78] Du W. J., Chi Y., Yang Z. X. (2016). Heterogeneity of proangiogenic features in mesenchymal stem cells derived from bone marrow, adipose tissue, umbilical cord, and placenta. *Stem Cell Research & Therapy*.

[79] Natan D., Nagler A., Arav A. (2009). Freeze-drying of mononuclear cells derived from umbilical cord blood followed by colony formation. *PLoS One*.

[80] Komaki M., Numata Y., Morioka C. (2017). Exosomes of human placenta-derived mesenchymal stem cells stimulate angiogenesis. *Stem Cell Research & Therapy*.

[81] Koob T. J., Rennert R., Zabek N. (2013). Biological properties of dehydrated human amnion/chorion composite graft: implications for chronic wound healing. *International Wound Journal*.

[82] Markov A., Thangavelu L., Aravindhan S. (2021). Mesenchymal stem/stromal cells as a valuable source for the treatment of immune-mediated disorders. *Stem Cell Research & Therapy*.

[83] Farsalinos K., Barbouni A., Niaura R. (2020). Systematic review of the prevalence of current smoking among hospitalized COVID-19 patients in China: could nicotine be a therapeutic option?. *Internal and Emergency Medicine*.

[84] Wong J. P., Viswanathan S., Wang M., Sun L. Q., Clark G. C., D'Elia R. V. (2017). Current and future developments in the treatment of virus-induced hypercytokinemia. *Future Medicinal Chemistry*.

[85] Liu Q., Zhou Y. H., Yang Z. Q. (2016). The cytokine storm of severe influenza and development of immunomodulatory therapy. *Cellular & Molecular Immunology*.

[86] Mogensen T. H., Paludan S. R. (2001). Molecular pathways in virus-induced cytokine production. *Microbiology and Molecular Biology Reviews*.

[87] Costela-Ruiz V. J., Illescas-Montes R., Puerta-Puerta J. M., Ruiz C., Melguizo-Rodríguez L. (2020). SARS-CoV-2 infection: the role of cytokines in COVID-19 disease. *Cytokine & Growth Factor Reviews*.

[88] Zhang C., Wu Z., Li J. W., Zhao H., Wang G. Q. (2020). Cytokine release syndrome in severe COVID-19: interleukin-6 receptor antagonist tocilizumab may be the key to reduce mortality. *International Journal of Antimicrobial Agents*.

[89] Chen K., Kolls J. K. (2016). Innate lymphoid cells and acute respiratory distress syndrome. *American Journal of Respiratory and Critical Care Medicine*.

